# Clinical significance of prostate volume and testosterone reduction on lower urinary tract symptoms in patients with prostate cancer undergoing androgen deprivation therapy

**DOI:** 10.1038/s41598-022-21963-1

**Published:** 2022-11-02

**Authors:** Woo Jin Bang, Hwanik Kim, Cheol Young Oh, Jung Ki Jo, Jin Seon Cho, Myungsun Shim

**Affiliations:** 1 Department of Urology, Hallym University College of Medicine, Hallym University Sacred Heart Hospital, 22, Gwanpyeong-ro 170 beon-gil, Dongan-gu, Anyang, Gyeonggi-do 14068 South Korea; 2 Department of Urology, College of Medicine, Hanyang University, Seoul, South Korea

**Keywords:** Urological cancer, Urology

## Abstract

To investigate the effect of both prostate volume and serum testosterone changes on lower urinary tract symptoms in patients with prostate cancer undergoing androgen deprivation therapy. A total of 167 patients who received androgen deprivation therapy for prostate cancer treatment from January 2010 to August 2020 were enrolled in this retrospective study. Changes in the International Prostate Symptom Score (IPSS) in the patient groups stratified by prostate volume and the amount of testosterone reduction were assessed every 4 weeks until 12 weeks after androgen deprivation therapy initiation. Longitudinal mixed models were used to assess the adjusted effects of prostate volume and testosterone reduction on IPSS change. All mean values of IPSS-total score (IPSS-total), voiding subscore (IPSS-vs), and storage subscore (IPSS-ss) significantly decreased from baseline to week 12 in both patients with small (< 33 mL) and large (≥ 33 mL) prostates. The mean values of IPSS-total, IPSS-vs, and IPSS-ss similarly decreased in patients with large prostate with a baseline IPSS-total of ≥ 13. However, in those with small prostate, IPSS-ss specifically remained unchanged, while IPSS-total and IPSS-vs significantly decreased. In addition, only in patients with small prostate (< 33 mL), patients with lesser testosterone reduction (< Δ400 ng/dL) showed greater improvement in IPSS-ss by 7.5% compared with those with greater testosterone reduction (≥ Δ400 ng/dL). In conclusion, although androgen deprivation therapy generally improves lower urinary tract symptoms, it may worsen specifically storage symptoms in patients with relatively small prostate and greater testosterone reduction. Our finding suggests that testosterone may influence lower urinary tract symptoms in these patients.

## Introduction

Lower urinary tract symptoms (LUTS) usually do not appear until the tumor has grown to compress or invade adjacent structures (e.g., the prostatic urethra, urinary bladder, or neurovascular bundles) because the majority of prostate cancer (PCa) arises in the peripheral zone of the prostate^1,2^. However, patients with early PCa may also develop LUTS, as the prevalence of benign prostatic hyperplasia increases with age, as does those of PCa. Overall, almost 45% of patients with PCa suffer from moderate to severe LUTS^3–5^.

In recent years, there have been reports that ADT performed as neoadjuvant therapy before radiation therapy or brachytherapy may significantly reduce prostate volume (PV)^6,7^. Thus, the relationship between ADT and LUTS relief has been extensively studied because ADT reduces prostate size. Reports exist on cases in which patients with PCa who experienced acute urinary retention were able to regain the ability to void after ADT^8^. On the other hand, the association between ADT and LUTS may be due not only to ADT reducing the PV but also due to changes in circulating testosterone. A previous study found that low testosterone levels in patients with bladder outlet obstruction negatively correlated with detrusor pressure while enhancing detrusor overactivity^9^. Thus, sex steroids in the genitourinary tract not only regulate the development and function of sex organs but may also influence lower urinary tract function^10^. Both epidemiological and experimental studies have revealed a link may exist between testosterone and volume threshold for inducing micturition^11,12^. However, previous and current studies have solely focused on the correlation between PV reduction and LUTS improvement in patients with PCa undergoing ADT, although testosterone may affect micturition.

Therefore, in this study evaluated the combined effect of PV and testosterone reduction on LUTS in patients with PCa undergoing ADT by analyzing the International Prostate Symptom Score (IPSS) changes stratified by the amount of both PV and serum testosterone reduction.

## Results

### Baseline characteristics

Patient baseline characteristics were generally similar between the groups stratified by PV. However, patients with large prostate (≥ 33 mL) demonstrated a higher mean IPSS-total score (IPSS-total) and IPSS voiding subscore (IPSS-vs) (all p < 0.050) compared with those with a relatively small prostate (< 33 mL), whereas the difference was not significant regarding IPSS storage subscore (IPSS-ss) (p = 0.078). Smaller FBC and lower Qmax were also noted in patients with large prostate when compared with those of small prostate (182.35 ± 74.31 vs 303.46 ± 122.78, p = 0.023 and 8.98 ± 2.88 vs. 17.92 ± 6.11, p < 0.001, respectively). Patient characteristics according to PV are shown in Table 1.

**Table 1:** Patient characteristics according to prostate volume.

Variable	Total	Prostate volume		*p*
	(N = 167)	< 33 mL (N = 81)	≥ 33 mL (N = 86)	
Age (years)	73.43 ± 7.12	72.99 ± 6.88	73.84 ± 7.36	0.589
BMI (kg/m^2^)	23.91 ± 2.83	23.58 ± 2.82	24.22 ± 2.83	0.678
PSA (ng/mL)	150.41 ± 264.86	163.66 ± 300.89	138.51 ± 225.37	0.035
Baseline testosterone (ng/dL)	429.02 ± 199.13	425.23 ± 159.62	433.16 ± 232.57	0.589
**Testosterone reduction (%)**				0.824
< Δ400 ng/dL	56 (33.5)	27 (33.3)	29 (33.7)	
≥ Δ400 ng/dL	111 (66.5)	54 (66.7)	57 (66.3)	
Prostate volume (mL)	36.51 ± 20.28	25.99 ± 4.47	46.42 ± 24.08	< 0.001
**Gleason score (%)**				0.105
6	6 (3.6)	2 (2.5)	4 (4.6)	
7	52 (31.1)	27 (33.3)	25 (29.1)	
8–10	109 (65.3)	52 (64.2)	57 (66.3)	
**Tumor stage**				0.617
T1/2	33 (19.8)	15 (19.8)	18 (21.0)	
T3/4	134 (80.2)	66 (80.2)	68 (79.0)	
Distant metastasis	141 (84.4)	72 (88.8)	69 (80.2)	0.237
**LHRH agonist type (%)**				0.128
Goserelin	78 (46.7)	38 (46.9)	37 (43.0)	
Triptorelin	59 (35.3)	26 (32.1)	36 (41.9)	
Leuprolide	30 (18.0)	17 (21.0)	13 (15.1)	
Oral bicalutamide	85 (50.9)	45 (55.5)	40 (46.5)	0.315
**IPSS**				
Total score	17.01 ± 7.49	12.04 ± 5.64	21.68 ± 5.83	< 0.001
Voiding subscore	9.33 ± 4.88	5.43 ± 2.48	13.01 ± 3.54	< 0.001
Storage subscore	7.68 ± 4.01	6.61 ± 4.21	8.68 ± 3.59	0.078
QoL score	3.30 ± 1.30	2.77 ± 1.25	3.81 ± 1.14	0.057
Functional bladder capacity (mL)	241.09 ± 117.38	303.46 ± 122.78	182.35 ± 74.31	0.023
*Q*_max_ (mL/s)	13.32 ± 6.50	17.92 ± 6.11	8.98 ± 2.88	< 0.001
Postvoid residual urine (mL)	35.11 ± 31.59	31.19 ± 32.65	38.80 ± 30.28	0.089

Values are presented as mean ± standard deviation or number (in percentage).

*BMI* body mass index, *PSA* prostate-specific antigen, *ADT* androgen deprivation therapy, *LHRH* luteinizing hormone-releasing hormone, *IPSS* International Prostate Symptom Score, *Q*_*max*_ maximal uroflow rate.

### IPSS changes according to PV

**Figure 1:**
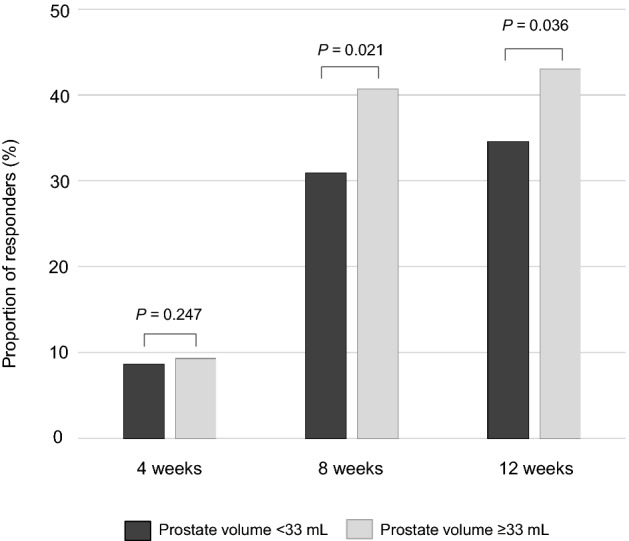
The proportion of patients showing clinically meaningful response (responders) defined as an International Prostate Symptom Score reduction of at least three points after androgen deprivation therapy.

A significantly higher proportion of patients showing clinically meaningful LUTS relief (responders) were noted in patients with large prostate compared with small prostate after 8 weeks. This trend continued until week 12 (40.7% vs. 30.9%, p = 0.021 and 43.0% vs. 34.6%, p = 0.036, respectively, Fig. 1).

**Figure 2:**
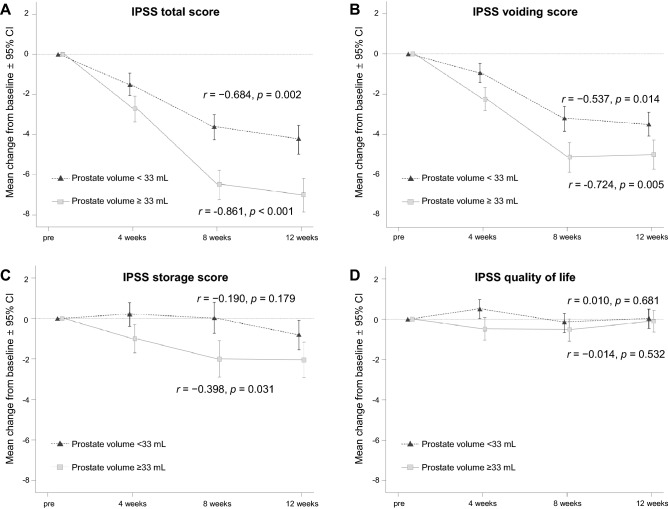
Mean (± standard error) changes in the International Prostate Symptom Score (IPSS) from baseline to 12 weeks after androgen deprivation therapy in patients with baseline IPSS of ≥ 13, stratified by prostate volume (mL). (**A**) IPSS total score; (**B**) IPSS voiding score; (**C**) IPSS storage score; (**D**) IPSS quality of life score.

All mean values of IPSS-total, IPSS-vs, and IPSS-ss significantly decreased from baseline to week 12 in both patients with small (r =  − 0.563, − 0.485, and − 0.232, all p < 0.050, respectively) and large (r =  − 0.759, − 0.626, and − 0.357, all p < 0.050, respectively) prostates. Focusing on patients with a baseline IPSS-total of ≥ 13 (a commonly used threshold in studies on the management of LUTS), IPSS-total, IPSS-vs, and IPSS-ss similarly significantly decreased in patients with large prostate (r =  − 0.861, − 0.724, and − 0.398, all p < 0.050, respectively, Fig. 2A–C). However, although the decreasing trend of IPSS-total and IPSS-vs (r =  − 0.684 and − 0.537, all p < 0.050, respectively, Fig. 2A,B) was similar with those in patients with large prostate, IPSS-ss remained virtually unchanged from baseline to week 12 (r =  − 0.190, p = 0.179, Fig. 2C) in patients with small prostate. No significant change was noted in the quality of life (QoL) score throughout the 12-week period both in patients with large and small prostate (Fig. 2D).

### Significance of testosterone reduction on IPSS change

Focusing on the patients with a baseline IPSS-total of ≥ 13, no difference exists in the total patients and those with large prostate in the magnitude of changes in all four IPSS categories after 12 weeks of ADT (IPSS-total, IPSS-vs, IPSS-ss, and QoL score; all p > 0.050) between patient groups stratified by the amount of serum testosterone reduction from baseline to week 12 (Δ serum testosterone, < Δ400 vs. ≥ Δ400 ng/dL, Table 2). However, no significant differences exist in changes in IPSS-total, IPSS-vs, and QoL score in patients with small prostate, whereas a greater decrease was noted in IPSS-ss in the group with smaller testosterone reduction (p = 0.037, Table 2). In patients with serum testosterone reduction of ≥ Δ400 ng/dL, a 0.08 ± 0.46 increase was noted in the mean IPSS-ss value in 12 weeks after ADT.

**Table 2:** Comparison of changes in International prostate symptom score (IPSS) from baseline to 12 weeks after androgen deprivation therapy according to serum testosterone reduction (Δ) stratified by prostate volume in patients with baseline IPSS of ≥ 13.

Change in clinical parameters	Serum testosterone reduction (ng/dL)		*p*
	< Δ400 ng/dL	≥ Δ400 ng/dL	
Prostate volume < 33 mL	N = 16	N = 23	
Δ IPSS total score	− 4.85 ± 2.35	− 3.84 ± 2.76	0.087
Δ IPSS voiding subscore	− 3.45 ± 1.97	− 3.58 ± 1.73	0.213
Δ IPSS storage subscore	− 1.85 ± 0.45	0.08 ± 0.46	0.037
Δ QoL score	− 0.22 ± 0.09	0.17 ± 0.21	0.478

Prostate volume ≥ 33 mL	N = 36	N = 45	
Δ IPSS total score	− 7.09 ± 2.94	− 6.98 ± 3.21	0.758
Δ IPSS voiding subscore	− 4.99 ± 3.17	− 5.11 ± 2.14	0.617
Δ IPSS storage subscore	− 2.28 ± 1.02	− 1.85 ± 0.71	0.102
Δ QoL score	− 0.11 ± 0.12	− 0.10 ± 0.34	0.813

Values are presented as mean ± standard deviation.

*IPSS* International Prostate Symptom Score, *QoL* quality of life.

### Adjusted effect of PV and serum testosterone on IPSS

Adjusted and unadjusted longitudinal models demonstrated modest, positive effects of PV on LUTS improvement with a 9.5% greater IPSS-total and 12.5% greater IPSS-vs decrease (p = 0.026 and p = 0.003, respectively) in patients with large (≥ 33 mL) prostates compared with those with small (< 33 mL) prostates over time (Table 3). No significant effects of PV on IPSS-ss and QoL score improvement were noted (all p > 0.050, Table 3). In longitudinal mixed models specifically performed in patients with PV of < 33 mL (n = 81), patients with lesser serum testosterone reduction (< Δ400 ng/dL) showed 7.5% greater IPSS-ss improvement (p = 0.038, Table 3) compared with those with greater serum testosterone reduction (≥ 400 ng/dL) over time. However, there is no significant effect of serum testosterone reduction on IPSS-total, IPSS-vs, and QoL score (all p > 0.050, Table 3).

**Table 3:** Change from baseline International Prostate Symptom Score after androgen deprivation therapy evaluated by longitudinal mixed modeling of baseline prostate volume and serum testosterone reduction.

	Unadjusted		Age-adjusted		Fully adjusted	
	% change per unit (95% CI)	*p*	% change per unit (95% CI)	*p*	% change per unit (95% CI)	*p*
**Total score**						
Prostate volume (< 33 vs. ≥ 33 mL)	10.0 (3.2, 17.6)	< 0.001	10.5 (4.3, 15.7)	0.015	9.5 (3.5, 15.8)	0.026
Serum testosterone reduction (< **Δ**400 vs. ≥ **Δ**400 ng/dL)*	3.4 (− 3.0, 8.8)	0.327	3.9 (− 2.4, 10.3)	0.389	3.8 (− 2.6, 9.6)	0.329
**Voiding subscore**						
Prostate volume (< 33 vs. ≥ 33 mL)	15.5 (5.6, 22.0)	< 0.001	13.3 (3.3, 21.5)	< 0.001	12.5 (3.0, 22.7)	0.003
Serum testosterone reduction (< **Δ**400 vs. ≥ **Δ**400 ng/dL) *	2.1 (− 4.6, 9.3)	0.238	2.4 (− 4.1, 9.6)	0.237	2.0 (− 3.3, 7.8)	0.386
**Storage subscore**						
Prostate volume (< 33 vs. ≥ 33 mL)	3.7 (− 2.3, 9.5)	0.673	3.5 (− 2.5, 8.8)	0.671	3.0 (− 1.8, 8.5)	0.519
Serum testosterone reduction (< **Δ**400 vs. ≥ **Δ**400 ng/dL) *	7.2 (2.2, 12.2)	0.035	6.7 (1.2, 11.1)	0.019	7.5 (1.2, 12.4)	0.038
**QoL score**						
Prostate volume (< 33 vs. ≥ 33 mL)	2.5 (− 2.8, 7.5)	0.855	− 1.8 (− 5.7, 3.3)	0.617	2.0 (− 3.4,7.2)	0.738
Serum testosterone reduction (< Δ400 vs. ≥ Δ400 ng/dL)*	1.3 (− 2.3, 4.8)	0.851	− 1.3 (− 3.4, 1.9)	0.451	1.5 (− 1.4, 5.2)	0.617

Model covariates for adjustment: age, body mass index, prostate-specific antigen, Gleason score, TNM stage, luteinizing hormone-releasing hormone agonist type and oral bicalutamide.

*QoL* quality of life.

*Analyzed only in patients with prostate volume of < 33 mL.

## Discussion

The data in this study demonstrated that more patients showed clinically meaningful LUTS relief after 12 weeks of ADT in patients with large prostate (PV ≥ 33 mL) compared with small (PV < 33 mL) prostate (40.7% vs. 30.9%, respectively). Moreover, the improvement of IPSS-total and IPSS-vs was more prominent in patients with large prostate compared with those with small prostate (r =  − 0.861 vs. − 0.684 and r =  − 0.724 vs. − 0.537, respectively) after excluding patients with mild LUTS (IPSS 0–7) who were not candidates for medical intervention. Previous studies have also shown the consisting findings that PV decreased from 18 to 41% when ADT was administered for 3–8 months, theoretically suggesting that ADT can improve LUTS^13^. Moreover, baseline PV before ADT initiation positively correlated with percent volume reduction during ADT^14^, suggesting that patients with larger prostate may experience more PV reduction and thus improved LUTS.

In the previous studies, the relationship between testosterone and LUTS often disappeared after statistical adjustment for age^15^, indicating that testosterone may not be the primary cause of the anatomical and functional change in the urinary tract. Nevertheless, numerous studies focused on the interrelation between circulating testosterone levels and LUTS at the molecular level by discovering a large extent of androgen receptors at the urethra and bladder^16^. The role of testosterone and its metabolites on neuromuscular transmission was also noted in rat isolated urinary bladder^17^. In addition, testosterone exerts its ability by manipulating nitric oxide, which acts as a nonadrenergic-noncholinergic neurotransmitter in the urogenital tract^18^. The positive effects of testosterone, i.e., increased bladder capacity and compliance and decreased detrusor pressure, on urinary symptoms were observed in a clinical study^19,20^. The higher serum levels of testosterone generated by testosterone supplementation were associated with reduced IPSS, probably indicating a correlation between serum testosterone level and their effects on LUTS^21^. Especially, lower IPSS-ss were reported in patients with testosterone supplementation than control by a more recent study based on a large registry data^22,23^. In the same context, an Asian study that investigated the relationship between male overactive bladder (OAB) symptoms and androgen deprivation revealed that ADT in patients with PCa was associated with an increased risk of OAB that was ADT duration-dependent^23^. Taken together, considering that the changes in both PV and testosterone during ADT can affect LUTS, analyzing that the combined effect of the two may provide relevant suggestions to enlighten the actual cause of changes in LUTS in patients with PCa undergoing ADT.

Unlike previous studies that solely focused on the association between prostate size and LUTS improvement in patients with PCa undergoing ADT, the present study primarily aims to determine the effect of both baseline PV and serum testosterone reduction after ADT on LUTS changes over 12 weeks. Consistent with previous reports, more significantly reduced scores were observed in IPSS-total and IPSS-vs in patients with large prostate compared with those with small prostate. However, as for IPSS-ss, the score reduction was not significant specifically in patients with small prostate suggesting that storage symptom during ADT does not share the mechanism of voiding symptom improvement, which is due to the PV reduction. This point focused on the role of testosterone on LUTS, particularly storage symptoms. Thus, these results that analyzed the effect of testosterone on IPSS changes revealed that the decreased amount of testosterone and those of IPSS-ss were inversely correlated. In patients with baseline IPSS ≥ 13, PV < 33 mL, and a decrease in testosterone above 400 ng/dL after 12 weeks of ADT, IPSS-ss was rather increased by 0.08 ± 0.46 (Table 2), indicating that patients with small prostate and greater testosterone reduction are likely to experience storage symptoms to be not improved during ADT, compare to those with small prostate and lesser testosterone reduction. Although voiding symptoms are expected to be improved during ADT due to PV reduction effect, storage symptoms are reckoned to be the opposite due to the reduction effect of testosterone. Furthermore, serum testosterone reduction by ADT maintained its effect on storage symptoms after adjusting PV along with other covariates (e.g., age at ADT initiation, BMI, tumor characteristics, and LHRH agonist type). In this study, the relationship between testosterone and LUTS, which was not clearly shown in previous studies, was observed probably due to the methodology of subclassifying patients more specifically (e.g., prostate size, baseline IPSS, and amount of testosterone reduction after ADT).

This study should mention the following limitation. Although IPSS reduction was noted, the concomitant PV reduction was not confirmed as follow up PV measurement was not performed in this study. Therefore, the proportion of the improvement of symptoms was not confirmed as the size decreases. In addition, urodynamic parameters corresponding to IPSS measurements were not repeatedly checked, which may have made it possible to more specifically analyze the pattern of IPSS changes. Additionally, the results of serum testosterone levels measurement may not be consistent from patient to patient because it can vary depending on the time of measurement during a day. However, this study tried to keep the measurement time as constant as possible by limiting the time from 9:00 am and noon. The absence of a control group is another drawback in this analysis.

In conclusions, LUTS generally improves in patients with PCa undergoing ADT. However, in patients with clinically meaningful symptoms (IPSS-total ≥ 13), storage symptom did not improve significantly in those with small prostate (< 33 mL). Storage symptoms even worsened in patients with both small prostate and greatest testosterone reduction (> 470 ng/dL). Taken together, both PV and testosterone change during ADT can influence LUTS. In particular, changes in testosterone can specifically affect the storage symptoms.

## Methods

### Study overview and inclusion/exclusion criteria

The present study protocol was reviewed and approved by the Institutional Review Board of Hallym University Sacred Heart Hospital (approval No. 2020-12-012). All research was performed in accordance with relevant guidelines and regulations, including the Declaration of Helsinki. A waiver of informed consent was granted due to the retrospective nature of this study. A total of 167 patients with locally advanced or metastatic PCa, with an estimated life expectancy of at least 12 months, and undergoing ADT for > 3 months from January 2010 to August 2020 were included for evaluation. All patients received one of three luteinizing hormone-releasing hormone (LHRH) agonists (goserelin acetate, 11.34 mg; triptorelin acetate, 11.25 mg; or leuprolide acetate, 11.25 mg) subcutaneously injected into the abdominal wall, and oral bicalutamide (50 mg) was administered daily. None of the included patients underwent bilateral orchiectomy as a form of ADT. The exclusion criteria were shown in Table 4. Especially, those with inappropriate data (those with missing IPSS records at 4, 8, 12 weeks, serum testosterone values at 4, 12 weeks) and significant pyuria suggesting recent urinary tract infection were excluded.

**Table 4:** Exclusion criteria.

Exclusion criteria
**Treating or being treated with followings within the previous month or after inclusion for > 1 month**
5-α reductase inhibitors
α-adrenoreceptor blockers
**Medications affecting bladder function such as sedative-hypnotics, antidepressants, antipsychotics, and loop diuretics within 1 month**
**Urinary catheterization**
**Any prostate surgeries or radiation therapy before or after inclusion**
**Recent urinary tract infection (within 1 month)**
**Previous diagnosis of followings**
Neurological disorders requiring treatment
Other types of cancer
**Developed a castration-resistant prostate cancer within the study period**

### Baseline parameters

The baseline parameters of patients, including age at ADT initiation, body mass index (BMI), prostate-specific antigen, cancer characteristics (Gleason score and TNM stage), pre-ADT IPSS, functional bladder capacity (FBC), maximal uroflow rate (Qmax), and postvoid residual urine volume (measured by diagnostic ultrasound), were evaluated. Serum testosterone was measured between 9:00 am and noon. PV was measured by transrectal ultrasonography, following the previously mentioned protocol^24^.

### Outcome measurements

The IPSS questionnaire was obtained from the patients before ADT at baseline and at 4, 8, and 12 weeks after ADT initiation to assess the severity of LUTS and its changes during ADT. Serum testosterone was also rechecked at 12 weeks after ADT start. The number and proportion (in percentage) of patients showing clinically meaningful LUTS relief (responder), which was defined as an IPSS-total score reduction of at least three points were evaluated according to PV^13^. IPSS was classified into four categories and analyzed respectively: IPSS-total, IPSS-vs, IPSS-ss, and QoL score. IPSS change was especially examined for patients with a baseline IPSS-total of ≥ 13. In addition, outcome measurements stratified by the amount of testosterone reduction from baseline and baseline PV were performed to specifically investigate the impact of testosterone and PV on LUTS relief in patients undergoing ADT. The median value of 33 mL for PV was used to categorize the patients for the comparison. Testosterone reduction value of Δ400 ng/dL for patient categorization was also used for the same reason.

### Statistical analysis

The differences in continuous and categorical variables between the groups stratified by PV were analyzed using the independent t-test and chi-square test, respectively. The significance of changing trends of IPSS values was confirmed by calculating the Pearson’s correlation coefficient and using ordered logistic regression. IPSS changes from baseline were also analyzed by analysis of covariance. IPSS parameter changes during ADT by PV and serum testosterone accounting for repeated, correlated observations in the same patient throughout the study period were evaluated using longitudinal mixed-model analyses. The recorded IPSS values were log-transformed for comparison. Multivariable modeling was utilized using recorded baseline covariates to control for potential confounders. All covariates were inserted into single multivariable models, which predict each outcome, and those with a p ≤ 0.2 in each model were included as final covariates for the full-adjusted model. All statistical analyses were performed using the SAS® Software 9.3 (SAS Institute, Cary, NC, USA), and a p-value of < 0.050 was considered to indicate statistical significance.

## Data Availability

The datasets generated during and/or analysed during the current study are available from the corresponding author on reasonable request.
